# Comparisons and associations among anthropometric indices of first and second division and assistant soccer referees

**DOI:** 10.3389/fpsyg.2023.1149779

**Published:** 2023-10-10

**Authors:** Meysam Rostamzadeh Samarein, Mohammad Hossein Samanipour, Foad Asjodi, Pooya Shokati, Zanyar Fallahi, Thomas E. Brownlee, João Paulo Brito, Nicola Luigi Bragazzi, Rafael Oliveira

**Affiliations:** ^1^Faculty of Sport Sciences, Shahrood University of Technology, Shahrood, Semnan, Iran; ^2^Department of Sport Science, Imam Khomeini International University, Qazvin, Iran; ^3^Iran Football Medical Assessment and Rehabilitation Center (IFMARK), FIFA Medical Center of Excellence, Tehran, Iran; ^4^Department of Exercise Physiology, Central Tehran Branch, Islamic Azad University, Tehran, Iran; ^5^Department of Sport Injury, Faculty of Physical Education and Sport Sciences, Tehran University, Tehran, Iran; ^6^Research Institute for Sport and Exercise Sciences, Liverpool John Moores University, Liverpool, United Kingdom; ^7^Sports Science School of Rio Maior–Polytechnic Institute of Santarém, Rio Maior, Portugal; ^8^Life Quality Research Centre, Rio Maior, Portugal; ^9^Research Center in Sport Sciences, Health Sciences and Human Development, Vila Real, Portugal; ^10^Department of Mathematics and Statistics, Laboratory for Industrial and Applied Mathematics (LIAM), York University, Toronto, ON, Canada; ^11^Human Nutrition Unit, Department of Food and Drugs, University of Parma, Medical School, Parma, Italy

**Keywords:** anthropometry, football, body composition, human body, height, weight

## Abstract

**Introduction:**

Body composition is an important predictor of performance and a key component of health and physical fitness. Therefore, the purposes of this study were to compare soccer referees of the first and second divisions and field assistant referees from Iran and to analyze associations of a body shape index (ABSI), body adiposity index (BAI), abdominal volume index (AVI), body roundness index (BRI), conicity index (ConI), and body mass index (BMI) with body fat percentage (%BF).

**Methods:**

A total of 270 male soccer referees from the first (*n* = 124) and second (*n* = 72) divisions and assistant referees (*n* = 74) participated in this study. Skinfold thickness (measured at the chest, biceps, triceps, subscapular, abdominal, iliac crest, and front thigh), height, weight, hip circumference, and waist circumference were assessed to evaluate waist-to-height ratio (WHtR), %BF, and also ABSI, BRI, BAI, ConI, and AVI according to the ISAK protocol.

**Results:**

The main results indicated differences in WHR, WHtR, ABSI, BRI, AVI, ConI, and BF% with the assistant referees presenting higher values (*p* < 0.05). When considering the backward selection model, there were some associations with %BF in each group, specifically BMI, BAI, and ABSI in the first division; BMI, WHR, and ABSI in the second division; and BMI in the assistant referee group (all *p* < 0.05).

**Discussion:**

The present study did not confirm the hypothesis that the first-division referees presented better body composition-related variables than the second division or assistant referees. Instead, it showed that the assistant referees that participated in both divisions showed a tendency to higher values which suggests that the level of division is not a major factor when analyzing body composition.

## 1. Introduction

Anthropometry refers to the measurements of the human individual and has been used for identifying and understanding human physical variations (Ehrampoush et al., 2017). These measurements have simple, easy, and effective characteristics that make them the first choice for structuring nutritional evaluations and interventions. Meanwhile, body composition assessment is an attempt to simplify a process that is inherently complex (Chen et al., 2016).

Research in this field illustrates that body composition has become critically important in understanding human metabolism in terms of health and performance (Thibault et al., 2012). Specifically, a high body fat percentage (%BF) is strongly associated with low fitness levels in adults (Wang et al., 2010). Furthermore, loss of muscle mass and high amounts of adipose tissue are associated with higher comorbidity (diabetes type 2, cardiovascular diseases, and cancers) and mortality (Hruby et al., 2016) rates. Similarly, in athletes, the improvement of body composition-related variables, such as lean body mass (LBM), is associated with enhanced performance and better outcomes in several exercise tests (Chiarlitti et al., 2018). Due to the importance of %BF in athleticism, its assessment is warranted. Consequently, for those working with soccer referees, the improvement of %BF is important to achieve the high physical standards required when refereeing in modern football (Casajus et al., 2014). In soccer, referees are the professionals responsible for controlling if rules are being followed during official matches (Reilly and Gregson, 2006; Schenk et al., 2018; Laws of the game 20/21, 2020). Despite their importance, only recently, research has focused on the body composition variables of soccer referees (Petri et al., 2020). When looking at the assessment of body composition, there are different methods such as measuring skinfold thickness or bioelectrical impedance measurements (U. S. Department of Health and Human Services, National Institutes of Health, 1998; Aragon et al., 2017). Recently, it has been proposed that using skinfold thickness is an easy method offering reliable results (Kasper et al., 2021). Moreover, Ashwell et al. (2012) examined some alternative indices instead of those introduced by the World Health Organization (WHO) to measure body fat, e.g., waist-to-height ratio (WHtR), conicity index (CI), and body adiposity index (BAI). Following the assessment by previous studies in overweight adolescents, it was found that there was a stronger relationship between fat mass (FM) and WHtR than between other indices such as body mass index (BMI) and body shape index (ABSI). Therefore, wherever FM cannot be measured, WHtR would be a reliable alternative to measure %BF in youth and adults (Eissmann, 1996; Ashtary-Larky et al., 2018).

In soccer, it is reported that over one million referees officiate matches every week in official competitions from all around the world (Castagna et al., 2007). A recent systematic review conducted in European and South American competitions regarding external load showed that the total average distance covered by the referees was, on average, 10–13 km throughout the entire match (Castagna et al., 2004; Mallo et al., 2009), with high-speed running distances accounting for 7%−17% of the match. This variability has been attributed to the distinct high-speed thresholds (ranging from 13.0 to 19.8 km.h^−1^) adopted in published research (Weston et al., 2012), where the most predominant speed occurred below 13 km.h^−1^ (Birk Preissler et al., 2023). The work rate *per* match of soccer referees is dependent on various variables, including the league level (Castagna et al., 2007), and interrelated with the work rate of outfield players, with large associations between referees and total team distance covered at a high-speed run (Weston et al., 2011).

Moreover, another study by Preissler et al. (2021) showed that the maximum heart rate and maximum speed measured during official matches were ~170 bpm and 24 km.h^−1^, respectively. The same study found a duration and percentage distance of 18% and 19%, respectively, at zones ≥90 and ≤ 100% of the maximum heart rate, while the speed zone (<13 km.h^−1^) showed a duration and a percentage distance of 94 and 86%, respectively, during official matches. The previous findings showed that soccer is a demanding sport in terms of physical conditioning for soccer referees. Indeed, due to the intense physical and cognitive requirements of matches, attention to the anthropometric, physical, and cognitive abilities of referees is of high importance to achieve the highest performance level (Casajús and Gonzalez-Aguero, 2015). Unfortunately, a large portion of research on physical match demands in soccer referees comes from European leagues (e.g., English, Danish, and Italian) (Krustrup and Bangsbo, 2001; Castagna et al., 2004; Weston et al., 2010), while soccer referees in Asian leagues are less studied (Fernandes da Silva et al., 2022).

Nonetheless, some results mentioned before revealed higher heterogeneity because different levels of competition were evaluated (Birk Preissler et al., 2023). In this respect, another study analyzed the first vs. second-division soccer referees and found higher values for the second division over the first referees regarding repeated sprint ability. The study justified such results with the higher level of experience of first-division referees that probably used better anticipation and pitch positioning, improved decision-making skills, and economical movement patterns, which consequently decreased the need of producing more sprints during competition (Meckel et al., 2020). For the previous reasons, it is expected that body composition differences between the first and second divisions could exist although no studies could be found in this regard. On the other hand, in a study recruiting Spanish referees, no differences were found between the first- and second-division referees during the 2001–2012 pre-seasons (Casajús and Gonzalez-Aguero, 2015).

Based on the aforementioned anthropometric and body composition variables, the present study aimed to compare body composition indices among soccer referees of the first and second divisions and assistant referees who participated in both divisions from Iran. Moreover, the study also analyzed the relationships among the different indices. It was hypothesized that the first-division referees presented better body composition variables and different body composition indices presented associations with %BF.

## 2. Materials and methods

### 2.1. Experimental design

This study was designed as a cross-sectional study. All anthropometric measurements were performed during the 2019–2020 in-season (March–April) before the outbreak of the COVID-19 pandemic in Iran.

### 2.2. Participants

The participants of this study were 270 male referees (124 first-division referees with an age, mean (standard deviation), of 28.0 (3.2) years, a mass of 72.5 (4.7) kg, a height of 175.9 (4.3) cm, and 8 years of experience, 72 second-division referees with an age of 28.5 (3.5) years, a mass of 74.8 (5.1) kg, a height of 175.4 (4.3), and 11 years of experience, and 74 field assistance referees with an age of 29.1 (3.3) years, a mass of 72.9 (4.9) kg, a height of 175.8 (4.0) cm, and 11 years of experience from the Iranian Football Federation). The assistant referees participated both in the first and second divisions. All participants provided informed written consent prior to participation. This study was conducted in accordance with the Declaration of Helsinki and approved by the Sports Science Research Institute of Iran (IR.SSRC.REC.1399.062).

### 2.3. Anthropometric assessment

This study used the international standards of the International Society for the Advancement of Kinanthropometry (ISAK). Measurements of skinfold thickness were performed at seven sites around the body (biceps, triceps, subscapular, iliac crest, supraspinal, abdominal, anterior thigh, and medial calf) as well as of weight, height, and WHR (waist-circumference and hip-circumference). Skinfold calipers (Harpenden, UK) with a sensitivity of 10 g/mm pressure on the skin, an anthropometric meter (Lufkin W606PM, UK), a stadiometer (Jushi No. 26SM, China) with an accuracy of 0.1 cm, and a weight scale (Maxy No. 9388, China) accurate to 0.1 kg were employed. In accordance with the ISAK protocol, duplicate measures were taken at each site, and, where the technical error of measurement (TEM) was <5%, the mean value was reported, and where the TEM was >5%, a third measure was taken and the median value was reported (Gibson-Smith et al., 2020; Nobari et al., 2020). It should be noted that all anthropometric measurements were performed by a 4-year expert with level 2 of ISAK certification in this field. The subjects were instructed to refrain from strenuous exercise on the day before measurements and not to smoke and drink alcohol, tea, and coffee on the day of testing. They were also asked not to exercise for 3 h, leading them to the tests. All anthropometric measurements were executed in the morning to increase the reliability of the measurement (Rahmat et al., 2016). Table 1 presents the formulas of the indices.

**Table 1:** Formulas of the anthropometric indices.

**Formulas**	**Units**
$ABSI=\;\frac{wc}{BMI^{2/3}\;\times \;ht^{1/2}}$	(Wc), cm/(Ht), cm/(BMI) Kg/m^2^ (Krakauer and Krakauer, 2012)
$BRI=364.2-365.5\;\times \;\sqrt{1-\frac{{\left(\frac{Wc}{2\pi }\right)}^{2}}{{\left(0.5\;\times \;Ht\right)}^{2}}}$	(Wc), cm/Constant no. (pi = 3.14159), π/(Ht), m (Thomas et al., 2013)
$WHR=\;\frac{wc}{hc}$	(Wc), cm/(Hc), cm (Han et al., 1997)
$BAI=\;\frac{hc}{Ht^{1.5}}-18$	(Hc), cm/(Ht), m (Wc), cm/(WHR) (Bergman et al., 2011)
$AVI=\;\frac{2wc^{2}+0.7{\left(Wc-hc\right)}^{2}}{1,000}$	Valdez et al., 1993
$ConI=\;\frac{Wc}{0.109\;\times \;\sqrt{\frac{Wt}{Ht}}}$	(Wc), m/(Wt), Kg/(Ht), m (Valdez, 1991)
$WHtR=\;\frac{wc}{ht}$	(Wc), cm/(Ht), cm (Han et al., 1997)
*Body Density* = 1.112 − 0.00043499 (Δ*sf*)+0.00000055(Δ*sf*^2^)−0.00028826 (*age*) (Cowan, 2013) Δ*sf* = Σ* Chest, Midaxillary, Triceps, Subscapular, Abdomen, Suprailiac, Thigh* Age:Years	
$Body\;Fat\;\%=\frac{457}{Body\;density}-414\;$(Brodie and Slade, 1988)	

ABSI, A Body Shape Index; BRI, Body Roundness Index; ConI, Conicity Index; Wc, waist circumference; Ht, height; AVI, Abdominal Volume Index; BAI, body adiposity index; BMI, body mass index; Hc, hip circumference; WHR, waist-to-hip ratio; WHtR, waist-to-height ratio; Wt, weight.

### 2.4. Statistical analysis

Descriptive statistics were conducted: means and standard deviations were used to characterize variables for each group as well as coefficients of variation (CVs). Kolmogorov–Smirnov and the Levene tests were used to test the assumption of normality and homoscedasticity, respectively. Then, comparisons were made through one-way analysis of variance (ANOVA) among all groups of referees. The significance level considered for all tests was a two-tailed *p*-value of <0.05. In the case of multiple comparisons between different indices in the first, second, and assistant group divisions, the adjustment method of false discovery rate (FDR) correction (Benjamini–Hochberg procedure) was used. The *q*-value has also been calculated.

The eta-squared value was used to calculate the effect size (ES) for comparisons between groups and its value was used to determine the magnitude of the effect using the following rule of thumb: 0.01 indicates a small effect, 0.06 indicates a medium effect, and 0.14 indicates a large effect. For comparisons between two groups, the ES calculated relied on Cohen's d, and its magnitude of significance was interpreted as follows: <0.2 = trivial, 0.2–0.6 = small effect, 0.6–1.2 = moderate effect, 1.2–2.0 = large effect, and >2.0 = very large (Liang et al., 2019).

In general, the association of %BF with other variables was studied by using regression models. Basic assumptions for conducting regressions (heteroscedasticity, collinearity, or outliers) were checked. Tolerance collinearity statistics was used to verify multicollinearity. Backward selection (known also as backward elimination) was employed to achieve the highest association possible. In the best-implemented regression model, %BF was the dependent variable and BMI, and WHR, WHtR, ABSI, BRI, AVI, BAI, and ConI variables were the independent variables.

More in detail, based on the backward selection regression model, in the first step, all variables were entered into the regression model. Then, at each step, the most insignificant variable that had the least effect on %BF was removed from the regression model. This process continued as long as it was possible, and the regression model could retain and obtain one or more significant variables (the so-called stopping rule).

All the statistical analyses were performed using the commercial software “Statistical Package for Social Sciences” (SPSS) for Windows (Version 28.0, IBM Corp., Armonk, NY, USA).

## 3. Results

The study included 270 referees, 124 (50%) from the first division, 72 (25.24%) from the second division, and 74 (24.76%) from assistant referees (A division). Comparisons of the three groups are presented in Table 2.

**Table 2:** Comparison of BMI, WHT, WHtR, ABSI, BRI, AVI, BAI, CI, and %BF values broken down by soccer referee groups (first, second division, and assistant referees).

**Parameters**	**CV**	**Group**	**Mean (SD)**	**CI, 95%**	***P*-value**	**Eta-squared value**
Weight (kg)	6.8%	1st	73.02 (5.60)	(72.02, 74.02)	0.315	0.01
		2nd	74.02 (3.74)	(73.15, 74.90)		
		A	72.92 (4.98)	(71.76, 74.07)		
Height (cm)	2.4%	1st	175.71 (4.66)	(174.89, 176.54)	0.976	0.00
		2nd	175.78 (3.63)	(174.93, 176.63)		
		A	175.85 (4.07)	(174.91, 176.79)		
BMI (kg/m^2^)	7.7%	1st	23.69 (2.04)	(23.32, 24.05)	0.408	0.01
		2nd	23.98 (1.46)	(23.64, 24.32)		
		A	23.60 (1.74)	(23.19, 24.00)		
WHR	10.7%	1st	0.81 (0.10)	(0.79, 0.83)	<0.001^*^	0.07
		2nd	0.80 (0.07)	(0.78, 0.82)		
		A	90.86 (0.06)	(0.84, 0.87)		
WHtR	8.7%	1st	0.43 (0.04)	(0.42, 0.44)	0.005^*^	0.04
		2nd	0.42 (0.03)	(0.42, 0.43)		
		A	0.44 (0.03)	(0.44, 0.45)		
ABSI [WC/(BMI^2/3*^height^1/2^)]	17.0%	1st	0.010 (0.002)	(0.010, 0.011)	0.017^*^	0.03
		2nd	0.010 (0.001)	(0.009, 0.010)		
		A	0.011 (0.002)	(0.010, 0.011)		
BRI	27.9%	1st	2.16 (0.68)	(2.04, 2.28)	0.006^*^	0.04
		2nd	2.07 (0.53)	(1.94, 2.19)		
		A	2.38 (0.52)	(2.26, 2.50)		
AVI	17.1%	1st	11.48 (2.20)	(11.08, 12.59)	0.004^*^	0.04
		2nd	11.18 (1.75)	(10.77, 11.59)		
		A	12.21 (1.65)	(11.82, 12.59)		
BAI (kg/m^2^)	14.7%	1st	22.36 (3.57)	(21.72, 22.99)	0.033^*^	0.03
		2nd	22.09 (2.99)	(21.39, 22.80)		
		A	21.14 (2.69)	(20.51, 21.76)		
ConI (cm^3^/2·kg^−1/2^)	8.7%	1st	107.46 (10.32)	(105.62, 109.30)	<0.001^*^	0.05
		2nd	105.47 (8.79)	(103.40, 107.54)		
		A	111.18 (7.42)	(109.46, 112.89)		
%BF	46.4%	1st	8.65 (2.82)	(8.15, 9.15)	<0.001^*^	0.77
		2nd	7.92 (2.00)	(7.45, 8.39)		
		A	18.53 (2.40)	(17.97, 19.08)		

1st, first-division referees; 2nd, second-division referees; A, assistant referees; BMI, body mass index; WHR, waist-to-hip ratio; WHtR, waist-to-height ratio; ABSI, A Body Shape Index; BRI, Body Roundness Index; AVI, Abdominal Volume Index; BAI, Body Adiposity Index; ConI, Conicity Index; CV, coefficient of variation.

^*^P < 0.05.

Weight, height, and BMI did not differ between groups. WHR, WHtR, ABSI, BRI, AVI, and ConI indices showed a significant difference among groups but with a very small ES with the assistant referees showing higher values than other referee groups. %BF was also higher in the assistant referees, with a large effect.

In the BAI index, the results also showed a significant difference between the groups with the first-division referees presenting higher values than other referees' groups. In Table 3, group-by-group comparisons were presented.

**Table 3:** Multiple comparisons between different indices in the first, second, and assistant group divisions using the adjustment method of false discovery rate (FDR) correction (Benjamini–Hochberg procedure).

**Indices**	**Mean difference (1st vs. 2nd)**	***P*, *q*; ES (1st vs. 2nd)**	**Mean difference (1st vs. A)**	***P*, *q*; ES (1st vs. A)**	**Mean difference (2nd vs. A)**	***P*, *q*; ES (2nd vs. A)**
Weight (kg)	−1.002	0.530, 0.815; 0.20	0.106	>0.999, 0.999; 0.02	1.109	0.543, 0.815; 0.25
Height (cm)	−0.066	>0.999, 0.999; 0.02	−0.137	>0.999, 0.999; 0.03	−0.071	>0.999, 0.999; 0.02
BMI (kg/m2)	−0.293	0.833, 0.999; 0.16	0.087	>999, 0.999; 0.05	0.381	0.623, 0.999; 0.24
WHR	0.007	>0.999, 0.999; 0.07	−0.049	<0.001^*^, 0.002; 0.55	−0.056	<0.001^*^, 0.002; 0.79
WHtR	0.005	>0.999, 0.999; 0.13	−0.014	0.031^*^, 0.047; 0.37	−0.019	0.006^*^, 0.018; 0.60
ABSI [WC/(BMI^2/3*^ height^1/2^)]	0.00046	0.219, 0.329; 0.26	−0.00036	0.476, 0.476; 0.20	−0.00082	0.013^*^, 0.039; 0.54
BRI	0.097	0.834, 0.834; 0.15	−0.215	0.049^*^, 0.074; 0.34	−0.312	0.006^*^, 0.018; 0.59
AVI	0.300	0.899, 0.0.899; 0.15	−0.729	0.034^*^, 0.049; 0.36	−1.029	0.005^*^, 0.015; 0.61
BAI (kg/m^2^)	0.263	>0.999, 0.999; 0.08	1.220	0.030^*^, 0.090; 0.37	0.957	0.215, 0.323; 0.34
ConI (m^3/2^·kg ^−1/2^)	1.989	0.437, 0.437; 0.20	−3.717	0.019^*^, 0.028; 0.40	−5.706	<0.001^*^, 0.003; 0.70
%BF	0.733	0.151, 0.151; 0.29	−9.879	<0.001^*^, 0.001; 3.69	−10.611	<0.001^*^, 0.001; 4.80

The q-value has also been calculated.

1st, first-division referees; 2nd, second-division referees; A, assistant referees; AVI, Abdominal Volume Index; ABSI, A Body Shape Index; BMI, body mass index; BRI, Body Roundness Index; BAI, Body Adiposity Index; CI, Conicity Index; WHR, waist-to-hip ratio; WHtR, waist-to-height ratio.

^*^P < 0.05.

In Table 4, there was no significant effect between %BF and other variables. For this reason, the backward selection regression model was used for each division in Table 5.

**Table 4:** Associations between variables, BMI, BRI, BAI, WHR, WHtR, ABSI, AVI, and ConI with BF% by using a backward selection regression model.

**Division**	**Variable**	**Unstandardized coefficients**		**Standardized coefficients**	***T***	***P*-value**	**%95 Confidence interval for** ***B***	
		***B***	**SE**	**Beta**			**Lower**	**Upper**
1st (*R* 0.34, *R*^2^ 0.12)	Constant	20.535	45.618		0.450	0.653	−69.826	110.896
	BMI	0.573	1.703	0.414	0.336	0.737	−2.801	3.946
	BRI	5.703	10.088	1.380	0.565	0.573	−14.280	25.686
	BAI	.633	0.400	0.799	1.582	0.116	−0.159	1.424
	WHR	19.463	18.241	0.687	1.067	0.288	−16.668	55.595
	WHtR	−158.992	283.430	−2.384	−0.561	0.576	−720.413	402.429
	ABSI	1,613.399	1,421.067	1.115	1.135	0.259	−1,201.461	4,428.259
	AVI	1.543	2.333	1.204	0.661	0.510	−3.078	6.163
	CI	−0.314	1.140	−1.148	−0.276	0.783	−2.573	1.944
2nd (*R* 0.22, *R*^2^ 0.05)	Constant	63.095	58.520		1.078	0.285	−53.813	180.003
	BMI	−1.835	1.271	−1.337	−1.444	0.154	−4.374	0.704
	BRI	1.981	6.333	0.525	0.313	0.755	−10.671	14.632
	BAI	0.380	0.752	0.568	0.505	0.615	−1.122	1.881
	WHR^*^	19.798	37.342	0.744	0.530	0.598	−54.800	94.396
	ABSI	−1,232.750	1,919.792	−0.872	−0.642	0.523	−5,067.975	2,602.476
	AVI	0.894	2.275	0.781	0.393	0.695	−3.650	5.439
	CI	−0.354	0.852	−1.555	−0.415	0.679	−2.056	1.348
A (*R* 0.96, *R*^2^ 0.92)	Constant	10.118	27.279		0.371	0.712	−44.363	64.598
	BMI	0.786	0.903	0.571	0.871	0.387	−1.017	2.588
	BRI	1.805	6.215	0.395	0.290	0.772	−10.606	14.216
	BAI	0.176	0.442	0.197	0.398	0.692	−0.707	1.059
	WHR	6.936	18.971	0.189	0.366	0.716	−30.950	44.823
	WHtR	−32.156	156.697	−0.420	−0.205	0.838	−345.102	280.789
	ABSI	−235.503	716.756	−0.158	−0.329	0.744	−1,666.964	1,195.957
	AVI	0.447	1.347	0.307	0.332	0.741	−2.244	3.137
	CI	−0.115	0.609	−0.355	−0.188	0.851	−1.331	1.102

1st, first-division referees; 2nd, second-division referees; A, assistant referees; AVI, Abdominal Volume Index; ABSI, A Body Shape Index; BMI, body mass index; BRI, Body Roundness Index; BAI, Body Adiposity Index; CI, Conicity Index; WHR, waist-to-hip ratio; WHtR, waist-to-height ratio.

^*^In the results of the second division, WHtR has been removed from the regression model due to strong collinearity (Tolerance Collinearity Statistics <0.001) with other variables. Significance level at a *P*-value of <0.05.

**Table 5:** Regression model (backward selection) for achieving the highest association with BF%.

**Division**	**Variable**	**Unstandardized coefficients**		**Standardized coefficients**	***T***	***P*-value**	**%95 Confidence interval for** ***B***	
		***B***	**SE**	**Beta**			**Lower**	**Upper**
1st (*R* 0.29, *R*^2^ 0.09)	Constant	−31.531	17.342		−1.818	0.072	−65.870	2.809
	BMI	1.317	0.701	0.953	1.879	0.063	−0.071	2.705
	BAI	0.174	0.079	0.219	2.203	0.030^*^	0.018	0.330
	WHtR	−26.378	17.474	−0.396	−1.510	0.134	−60.979	8.223
	ABSI	1,588.114	726.719	1.098	2.185	0.031^*^	149.138	3,027.090
2nd (*R* 0.21, *R*^2^ 0.05)	Constant	33.740	16.634		2.028	0.046	0.539	66.941
	BMI	−1.415	0.846	−1.032	−1.673	0.099	−3.104	0.273
	BAI	0.377	0.239	0.563	1.577	0.119	−0.100	0.853
	WHR	20.152	11.608	0.757	1.736	0.087	−3.018	43.322
	ABSI	−1,653.909	939.470	−1.170	−1.760	0.083	−3,529.100	221.282
A (*R* 0.96, *R*^2^ 0.91)	Constant	−12.490	1.137		−10.980	<0.001	−14.757	−10.222
	BMI	1.314	0.048	0.955	27.346	<0.001^**^	1.219	1.410

(α = 0.1). ^*^P < 0.01; (α = 0.05). ^**^P < 0.05.

1st, first-division referees; 2nd, second-division referees; A, assistant referees; AVI, Abdominal Volume Index; ABSI, A Body Shape Index; BMI, body mass index; BRI, Body Roundness Index; BAI, Body Adiposity Index; CI, Conicity Index; WHR, waist-to-hip ratio; WHtR, waist-to-height ratio.

Table 5 shows that BAI and ABSI had a significant effect on %BF in the first-division referees (respectively, *B* = 0.174, Beta = 0.219, *P*-value = 0.030; *B* = 1588.1, Beta = 1.098, *P*-value = 0.031). If the level (α = 0.1) is considered, BMI had a significant effect on %BF (*P*-value <0.01). In the second-division referees, BMI, WHR, and ABSI variables with level (α = 0.1) can be considered to have a significant effect on %BF (*P*-value <0.01). In the assistant referees, BMI had a significant effect on %BF (*B* = 1.314, Beta = 0.955, *P*-value <0.001).

## 4. Discussion

The study aimed to compare soccer referees of three groups: first and second divisions and assistant referees from Iran and to correlate different body composition indices. Considering the comparison analysis, there were differences in WHR, WHtR, ABSI, BRI, AVI, CI, and BF%, with the assistant referees presenting higher values. Concerning the BAI values, they tended to be slightly higher for referees of the first division even though they did not achieve the significance threshold and exhibited a small ES. Between the first and second divisions, there were no differences in all measures, while weight, height, and BMI did not show significant differences among the three groups.

WHR and BRI enabled the prediction of both body fat and the percentage of visceral adipose tissue (Thomas et al., 2013; Swainson et al., 2017). According to the measured values, both %BF and AVI were higher in the assistant referees, as well as WHR. While the present research is based on the %BF and several indices, research on WHR has investigated related health aspects and its capability to predict metabolic syndrome and fatty liver (Motamed et al., 2016). Based on this, Motamed et al. (2016) indicated that there was a weak correlation between WHR and non-alcoholic fatty liver disease. However, the waist circumference, WHR, WHtR, and AVI were reported as the strongest anthropometric discriminators of metabolic syndrome (Aune et al., 2016; Fontela et al., 2017) which may be an indicator of less favorable physical fitness (Wang et al., 2018; Wu et al., 2021). On the other hand, considering previous studies (Aune et al., 2016; Motamed et al., 2016; Fontela et al., 2017; Wang et al., 2018; Wu et al., 2021), WHR, WHtR, and AVI seem to be less relevant in sports professionals.

The ConI is an index of abdominal obesity that was developed based on a model of geometric reasoning (Valdez, 1991) and proved to be sensitive and better than the waist-to-hip ratio as an indicator of risk for hyperlipidemia (Christakoudi et al., 2020). Despite the first and second referees in the present study did not reveal significant differences, the assistant referees showed differences. According to parameters such as weight, height, and WC, evidence of less mass tissue accumulation could be found at the abdominal level in the first and second referees (Valdez, 1991; Motamed et al., 2016). However, it was not clear why assistant referees presented higher values, which is suggested to be explored in future studies.

The ABSI, which represents an alternative index to the indices of abdominal obesity, has an important allometric component because it enables the identification of individuals with normal weight but with abdominal obesity (Christakoudi et al., 2020). No differences were found between the first- and second-division referees in this parameter, but assistant referees differed from the other categories of referees. This may also be related to anthropometric differences in height and in the quantity and pattern of body mass distribution since the allometric component has a strong influence on this index. Moreover, it is also important to note that ABSI was considered to have an effect on %BF in both the first and second groups, while this was not noted in the assistant referee group. The fact that there were no differences in BMI in the three categories may be due to the fact that when comparing the different indices, the distribution of body mass may not have a direct relationship with allometry. Assistant referees showed a higher value in the WHR and %BF; nonetheless, these values were classified as healthier [World Health Organization (WHO), 2021].

It could also be mentioned that individuals with normal weight and abdominal obesity can show metabolic alterations, while obese individuals without abdominal adiposity can remain “metabolically healthy.” Nevertheless, while general obesity is widely evaluated with BMI (Fontela et al., 2017), according to the WHO categories (Birk Preissler et al., 2023), there is no current consensus on how best to assess abdominal adiposity, and various anthropometric indices incorporating waist circumference have been proposed in the literature (Thomas et al., 2013; Fontela et al., 2017; Woolcott and Bergman, 2018; Ofstad et al., 2019).

In summary, although BMI was similar in the three categories, it did not allow us to discern the organic distribution of the adipose tissue. Nonetheless, BMI was revealed to have a significant influence on %BF in all groups. However, the highest BAI values in the first and second referees were incongruous with respect to the rest of the results. All other differences in anthropometric parameters were in line with what is expected for referees in the first and second divisions with greater physical demands during games, regarding an external load (Preissler et al., 2021).

Despite the robustness of the present findings, this cross-sectional study had some limitations such as the fact that it was not possible to determine cause-and-effect relationships. Moreover, body composition was assessed through non-considered reference methods but, according to a recent study, anthropometry can also be used for valid FM% estimations (Campa et al., 2023). In addition, nutritional habits (Sarkar et al., 2019; Afrifa et al., 2020) could probably help justify why assistant referees tended to have higher values. This is even more important to highlight because assistant referees included professionals who performed in both the first and second divisions which makes plausible the speculation that the division level is not the most important variable to consider. However, nutritional habits were not assessed.

Future studies can include other variables from other dimensions such as internal/external measures and interventions/training protocols while controlling for nutritional habits to provide knowledge about the variations over the full soccer season. In addition, women referees should also be included to provide comparisons between sexes.

Finally, the strengths of this study were related to the large sample size and comprehensive body composition characterization of soccer referees. We provided data from Iran, which did not show significant differences between the level of divisions, which is similar to previous research (Casajús and Gonzalez-Aguero, 2015).

## 5. Conclusion

The present study did not confirm the hypothesis that the first-division referees presented better body composition-related variables than the second division or the assistant referees. In fact, the first- and second-division referees presented similar values for all variables, while assistant referees showed higher values in WHR, WHtR, ABSI, BRI, AVI, CI, and %BF. Instead, the present study showed that the assistant referees that participated in both divisions showed a tendency to higher values which suggests that the level of division is not a major factor when analyzing body composition.

When considering the backward selection model, there were some associations with %BF in each group, specifically BMI, BAI, and ABSI in the first division; BMI, WHR, and ABSI in the second division; and BMI in the assistant referee group. However, given the above-mentioned shortcomings, further research in the field is urgently warranted.

## Data availability statement

The original contributions presented in the study are included in the article/supplementary material, further inquiries can be directed to the corresponding author.

## Ethics statement

The studies involving humans were approved by the IRBs of the Iranian Universities which took part into the study. The studies were conducted in accordance with the local legislation and institutional requirements. The participants provided their written informed consent to participate in this study.

## Author contributions

All authors listed have made a substantial, direct, and intellectual contribution to the work and approved it for publication.
